# Availability of suPAR in emergency departments may improve risk stratification: a secondary analysis of the TRIAGE III trial

**DOI:** 10.1186/s13049-019-0621-7

**Published:** 2019-04-11

**Authors:** Martin Schultz, Line J. H. Rasmussen, Thomas Kallemose, Erik Kjøller, Morten N. Lind, Lisbet Ravn, Theis Lange, Lars Køber, Lars S. Rasmussen, Jesper Eugen-Olsen, Kasper Iversen

**Affiliations:** 1 Department of Cardiology, Herlev and Gentofte Hospital, University of Copenhagen, Ringvej 75, 2730 Herlev, Denmark; 2 Department of Internal medicine and Geriatrics, Herlev and Gentofte Hospital, University of Copenhagen, Ringvej 75, 2730 Herlev, Denmark; 3 Clinical Research Centre, Amager and Hvidovre Hospital, University of Copenhagen, Kettegård Alle 30, 2650 Hvidovre, Denmark; 4 Department of Emergency Medicine, Herlev and Gentofte Hospital, University of Copenhagen, Herlev ringvej 75, 2730 Herlev, Denmark; 5 Department of Public Health, University of Copenhagen, Section of biostatistics, Øster Farimagsgade 5, 1014 Copenhagen, Denmark; 6 Center for Statistical Science, Peking University, No. 5 Yiheyuan Road Haidian District, Beijing, 100871 China; 7 Department of Cardiology, Rigshospitalet, University of Copenhagen, Blegdamsvej 9, 2100 Copenhagen, Denmark; 8 Department of Anaesthesia, Centre of Head and Orthopaedics, Rigshospitalet, University of Copenhagen, Blegdamsvej 9, 2100 Copenhagen, Denmark

**Keywords:** Emergency department, Triage, Risk stratification, Prognostic biomarkers, suPAR

## Abstract

**Introduction:**

Soluble urokinase plasminogen activator receptor (suPAR) is a prognostic and nonspecific biomarker associated with short-term mortality in emergency department (ED) patients. Therefore, the blood level of suPAR might be usable for identification of patients at high- and low risk, shortly after arrival at the ED. Here, we investigate the value of adding suPAR to triage and how this may impact on risk stratification regarding mortality.

**Methods:**

The analyses were performed on the TRIAGE III cohort. Patients were triaged in four groups: Red, Orange, Yellow, and Green. Outcome was all-cause mortality within seven days. Discriminative abilities of triage and suPAR on mortality were assessed using the area under the curve (AUC) for receiver operating characteristics (ROC) curves. A suPAR cut-off value was generated using the Youden’s index. Patients were subsequently reclassified one triage level up if the suPAR level was above this cut-off and one level down if the suPAR level was below that value.

**Results:**

The study included 4420 patients with an available triage category and suPAR measurement. suPAR was significantly better in predicting mortality than triage; AUC (95% confidence interval): 0.85 (0.80–0.89) vs. 0.71 (0.64–0.78), *P* < 0.001. Combining suPAR and triage yielded an AUC of 0.87 (0.82–0-93). The Youden’s cut-off of suPAR was 5.9 ng/mL and reclassified triage using this value resulted in a more accurate risk stratification regarding hospital admission and mortality.

**Conclusion:**

Addition of suPAR to triage potentially improves prediction of short-term mortality. Measurement of suPAR in relation to the triage process may allow a more accurate identification of ED patients at risk.

**Trial registration:**

Clinicaltrials.gov, NCT02643459. Registered 31 December 2015. https://clinicaltrials.gov/ct2/show/NCT02643459?cond=NCT02643459&rank=1.

**Electronic supplementary material:**

The online version of this article (10.1186/s13049-019-0621-7) contains supplementary material, which is available to authorized users.

## Background

Triage is the process of quickly assessing and prioritising patients according to urgency and need for treatment [1]. Most emergency departments (ED) use risk scoring systems to perform triage, [1, 2] and widely used conventional triage algorithms are 5-level scales relying on measurements of vital signs and the presenting complaint [1, 2]. Blood tests can also be included, [3, 4] and risk stratification models using various biomarkers have been shown to have high discriminative powers regarding mortality in patients arriving at the EDs [5, 6].

The protein soluble urokinase plasminogen activator receptor (suPAR) is a nonspecific biomarker that contains information on presence and severity of a broad variety of acute and chronic diseases. In addition, the suPAR level is associated with length of stay and transfer to the intensive care unit in patients presenting acutely to the EDs, as well as an independent predictor of short-term mortality [7–9]. Therefore, suPAR is a potential candidate to improve the accuracy in the triage process, however the impact of adding suPAR has not previously been explored.

The present study aimed to investigate if adding suPAR to conventional triage would improve accuracy prediction of mortality and how this may impact risk stratification.

## Methods

### Setting and design

The TRIAGE III trial was a cluster-randomised interventional trial investigating the effect of introducing suPAR as a routine biomarker in the ED. In this study, we performed post hoc secondary analyses investigating the effect of adding suPAR to triage in outcome prediction. The TRIAGE III trial collected data from an unselected population admitted to EDs at hospitals in Denmark. The two participating hospitals have large EDs with access to all medical specialities, general, and orthopaedic surgery, as well as intensive care, and manage 70,000 and 85,000 annual patient visits. Both are university hospitals with a catchment population of approximately 425,000 and 480,000 respectively of mostly city areas. In interventional periods suPAR measurement was available within 30–120 min of arrival alongside routine blood tests at acute admission (analysis of suPAR took 23 min using point-of-care equipment). In control periods suPAR was unavailable. In the current study, we included patients’ first ED visit where both the suPAR level was determined and a triage category was recorded. The protocol and primary results have been published [10, 11].

The EDs use the conventional triage algorithm “Danish Emergency Process Triage” (DEPT) [12], which consist of measurements of vital signs (blood pressure, heart rate, level of consciousness, arterial oxygen saturation, respiratory rate, and temperature), as well as an assessment of the presenting complaint. DEPT is a local variant of Adaptive process triage (ADAPT) [13]. The triage algorithm categorise patients into five groups: Red (most urgent), Orange, Yellow, Green (least urgent), and Blue (minor injuries) [14]. There were few patients triaged Blue (*N* = 103), and as they were similar to the patients in the Green category, Green and Blue categories were combined in these analyses.

### Analyses

Hospital admission was defined as a stay in the ED of more than 24 h or transfer from the ED to a stationary ward. This definition was necessary as patients arriving at the EDs are registered differently in the hospital records upon arrival (medical patients are registered as admitted even though some were discharged shortly after arrival). Length of stay were calculated in all hospital admissions. In the analysis, we sought to assess the ability to improve prediction of seven-day all-cause mortality by adding the suPAR level at arrival to the initial triage level. First, we assessed the discriminative ability of suPAR in predicting seven-day mortality using area under the curve (AUC) for receiver operating characteristics (ROC) curves.

Then, we identified a cut-off value of suPAR and used this threshold to reclassify patients one level up in triage category, if suPAR was above the threshold and one level down in triage category if the suPAR value was below the threshold.

### Statistics

Continuous variables are presented with median and interquartile range (IQR), or mean and standard deviation (SD) and compared using Wilcoxon Rank-sum test and Student’s two-sample t-test. Categorial variables are described as number (n) and percentage (%) and compared using chi-square test. The discriminative ability of triage and suPAR to predict seven-day all-cause mortality was analysed using AUC for ROC curves. Comparison of AUCs was done using the Delong method [15]. Sensitivity, specificity, positive predictive value (PPV), and negative predictive value (NPV) were calculated from cut-off based on the Youden’s index in a ROC analyses using a logistic regression [16]. Comparison of initial triage and reclassification was done using McNemar’s test and Fischer’s exact test. A *P*-value of < 0.05 was considered significant. Statistics were performed in R version 3.4.1 [17, 18].

## Results

Of 16,801 unique patients from the TRIAGE III population, the triage category was available in 9082 (54.1%) patients. In patients with triage categories 4420 (48.7%) arrived in interventional periods and had a suPAR level available, thus comprising the study population (Additional file 1: Figure S1). The mean age (SD) was 59.6 (20.8) years, and 2239 (50.7%) were women, and 2108 (47.7%) were admitted. The median (IQR) suPAR level was 3.9 ng/mL (2.9–5.8). At seven days 58 (1.3%) patients had died. Baseline characteristics are displayed in Table 1. Patients where no triage category was available were significantly older (61.6 vs 59.7 years, *P* < 0.001), more were women (55.5% vs 50.7, *P* < 0.001) and more were admitted (52.2% vs. 47.7%, *P* < 0.001), but there was no significant difference in all-cause mortality at seven days (1.6% vs 1.3%, *P* = 0.268).

**Table 1:** Baseline characteristics of patients at arrival at the emergency departments. Patients are grouped according to the initial triage category

Triage category *N* (%)	Included 4420 (100)	Red 215 (4.9)	Orange 1241 (28.1)	Yellow1,243 (28.1)	Green 1721 (38.9)
Female sex, *N* (%)	2239 (50.7)	92 (42.8)	643 (51.8)	602 (48.4)	902 (52.4)
Age, years, mean (SD)	59.7 (20.8)	62.3 (21.5)	63.5 (19.2)	60.5 (20.7)	55.9 (21.3)
Albumin, (g/L), median (IQR)	39 (35–43)	36 (32–40)	37 (33–40)	39 (34–43)	41 (38–44)
CRP (mg/L), median (IQR)	4 (3–32)	5 (3–33)	3 (3–23)	5 (3–35)	4 (3–41)
suPAR, ng/mL, median (IQR)	3.9 (2.9–5.8)	5.6 (3.4–7.1)	4.1 (2.9–6.0)	3.9 (2.9–5.9)	3.8 (2.8–5.4)
suPAR, patients alive at seven days, ng/mL, median (IQR)	3.9 (2.9–5.7)	4.8 (3.2–6.5)	4.1 (2.9–6.0)	3.9 (2.9–5.8)	3.8 (2.8–5.4)
suPAR, patients dead at seven days, ng/mL, median (IQR)	8.6 (6.0–12.8)	7.9 (5.9–9.7)	10.9 (8.1–15.0)	7.9 (6.3–11.1)	9.8 (5.3–14.7)
Cancer, *N* (%)	166 (3.8)	9 (4.2)	41 (3.3)	46 (3.7)	70 (4.1)
Cardiovascular disease, *N* (%)	956 (21.6)	79 (36.7)	419 (33.8)	239 (19,2)	219 (12.7)
Infectious disease, *N* (%)	830 (18.8)	50 (23.3)	207 (16.7)	228 (18.3)	345 (20.0)
Neurological disease, *N* (%)	341 (7.7)	27 (12.6)	117 (2.6)	99 (8.0)	98 (5.7)
General surgery, *N* (%)	168 (3.8)	6 (2.8)	26 (2.1)	54 (4.3)	82 (4.8)
Orthopaedic surgery, *N* (%)	154 (3.5)	1 (0.5)	102 (8.2)	26 (2.1)	25 (1.5)
Other, *N* (%)	1805 (40.8)	43 (20.0)	329 (26.5)	551 (44.3)	882 (51.2)
Herlev Hospital, *N* (%)	2780 (62.9)	85 (3.1)	628 (22.6)	683 (24.6)	1384 (49.8)
Bispebjerg Hospital, *N* (%)	1640 (37.1)	130 (8.0)	613 (37.3)	560 (34.2)	337 (20.5)
Hospital admission, *N* (%)	2108 (47.7)	143 (66.5)	706 (56.9)	615 (49.5)	644 (37.4)
Length of stay (days), mean (SD)	4.2 (7.1)	7.0 (11.1)	5.1 (7.9)	4.1 (6.2)	3.3 (6.2)
Surgery during admission, *N* (%)	444 (10.0)	15 (7.0)	164 (13.2)	112 (9.0)	153 (8.9)
Readmissions at seven days, *N* (%)	238 (5.4)	10 (4.7)	72 (5.8)	72 (5.8)	84 (4.9)
Mortality at seven days, *N* (%)	58 (1.3)	17 (7.9)	20 (1.6)	12 (1.0)	9 (0.5)

*CRP* C-Reactive Protein, *IQR* Interquartile range, *SD* standard deviation, *suPAR* Soluble urokinase plasminogen activator receptor

The number of patients in the triage categories were: Red: 215 (4.9%), Orange: 1241 (28.1%), Yellow: 1243 (28.1%), and Green: 1721 (38.9%). The suPAR level increased with more urgent triage categories and was significantly higher in non-survivors compared to survivors across all categories (*P* < 0.001).

**Fig. 1:**
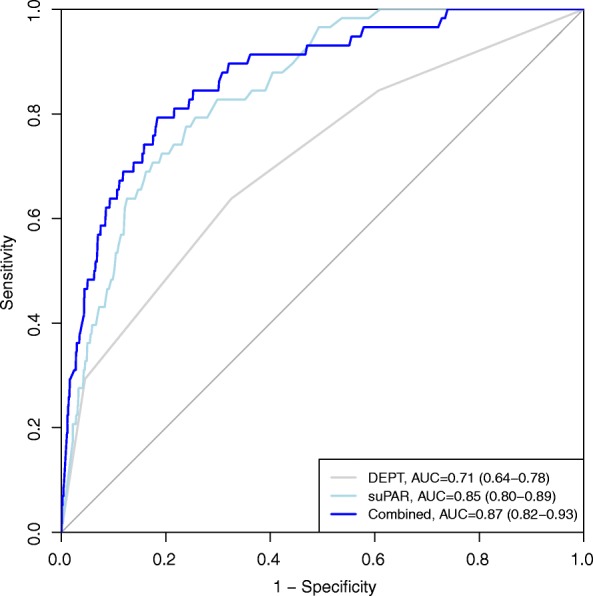
Area under the curve for discrimination on all-cause mortality at seven days for Danish Emergency Proces Triage (DEPT), soluble urokinase plasminogen activator receptor (suPAR) and the combination. DEPT vs suPAR. *P* < 0.001, DEPT vs Combined: *P* < 0.001, suPAR vs. Combined: *P* = 0.016

**Fig. 2:**
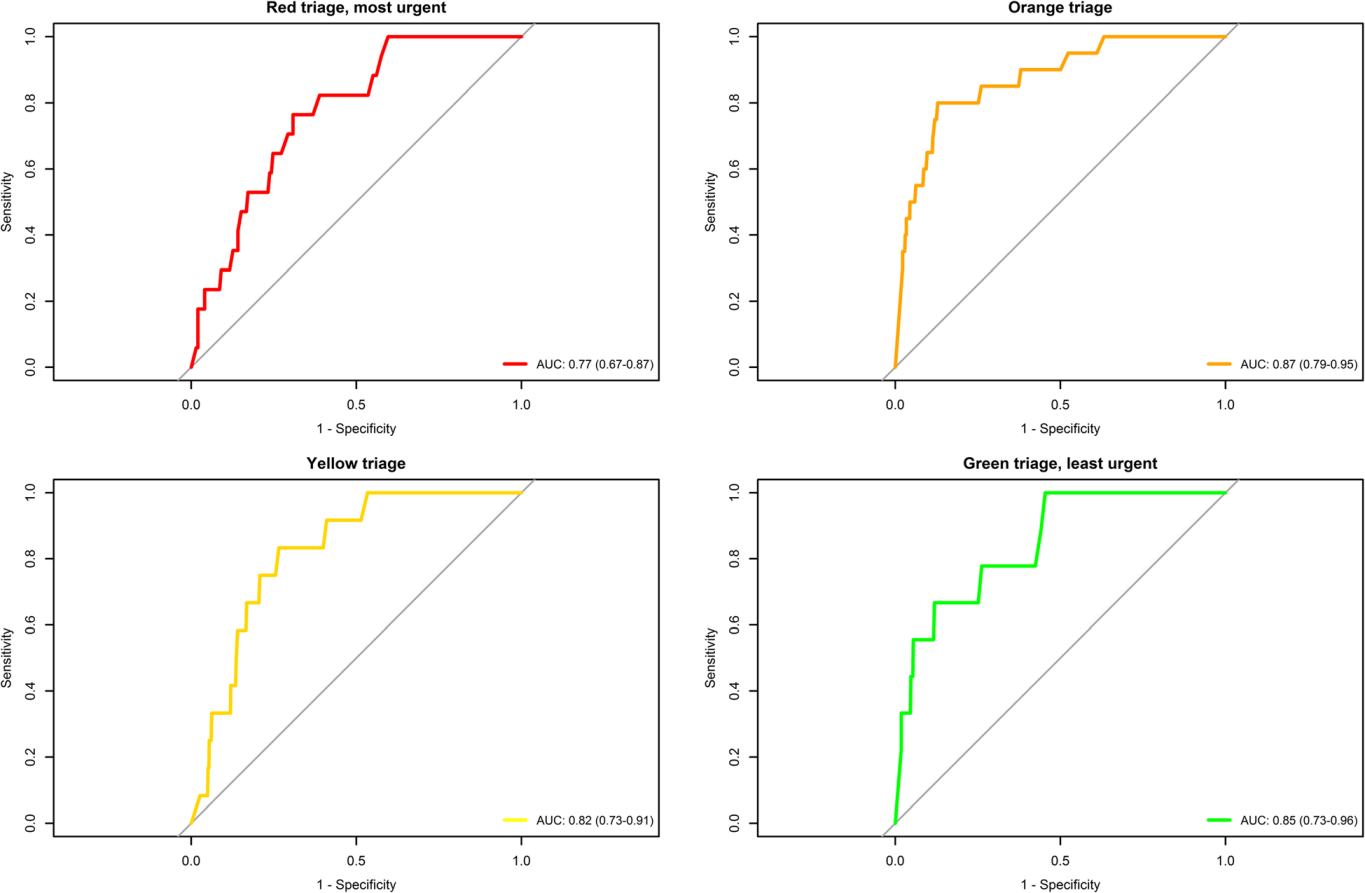
Predictive ability of suPAR in discriminating all-cause mortality at seven days. Patients arriving in two emergency departments stratified according to the triage level at arrival. Mortality in each group; Red: 17/215 (7.9%), Orange: 20/1241 (1.6%), Yellow: 12/1243 (1.0%), Green: 9/1721 (0.5%). suPAR: soluble urokinase plasminogen activator receptor

Discriminative abilities of suPAR in predicting seven-day mortality in all patients were significantly higher than DEPT triage; AUC (95% CI): 0.85 (0.80–0.89) vs. 0.71 (0.64–0.78), *P* < 0.001. In comparison, age had a AUC of 0.78 (0.74–0.83). Combining suPAR and DEPT yielded an AUC of 0.87 (0.82–0.93), which was not significantly higher than suPAR alone (*P* = 0.16), Fig. 1. A model with the addition of both suPAR and age to DEPT were not significantly better than suPAR alone (P = 0.16) or suPAR and DEPT (*P* = 0.10). suPAR had consistently high discriminative ability for seven-day mortality in all triage groups. (Fig. 2). The AUC for predicting hospital admission for DEPT was 0.60 (0.58–0.61) compared to the combination of DEPT and suPAR: 0.86 (0.82 to 0.91), *P* < 0.01. The optimal cut-off (threshold) for discriminating between survivors and non-survivors among all patients was 5.9 ng/mL (specificity: 0.77, sensitivity: 0.79, PPV: 0.05, NPV: 1.0). In the study population 3372 (76.3%) patients had a suPAR level below the threshold at arrival to the ED. Sensitivity analyses showed that a cut-off ranging from 5.5 ng/mL to 6.4 ng/mL performed equally well and yielded qualitatively same results.

Reclassification into new triage categories was then done according to a suPAR level above or below 5.9 ng/mL, Table 2. The reclassified triage had significantly more patients in the most urgent triage category (Red) compared to DEPT (9.0% vs. 4.9%, *P* < 0.001), significantly less patients in the Orange category (10.1% vs 28.1% *P* < 0.001), same number of patients in the Yellow (*P* = 0.968), and finally significantly more patients classified in the least urgent triage (Green) category compared to DEPT (52.3% vs. 38.9%, *P* < 0.001). The patients allocated to the Green category in the reclassified triage were significantly younger (*P* = 0.002) compared to DEPT, while the patients in the remaining categories were significantly older in the reclassified triage (Yellow: *P* = 0.002, Orange: *P* < 0.001, Red: *P* < 0.001), Table 2.

**Table 2:** Reclassification of triage categories according to the suPAR level measured at arrival

Triage category *N* (%)	Red 396 (9.0)^a^	Orange 445 (10.1)^a^	Yellow 1.267 (28.7)	Green 2312 (52.3)^a^
Female sex, *N* (%)	209 (52.8)	202 (45.4)	665 (52.5)	1163 (50.3)
Age, years, mean (SD)	74.8 (14.6)^a^	67.6 (20.0)^a^	62.8 (19.0)^a^	53.8 (20.6)^a^
Hospital admission, *N* (%)	324 (81.8)^a^	304 (68.3)^a^	665 (52.5)	815 (35.2)
Length of stay (days), mean (SD)	8.5 (11.8)	6.9 (9.5)^a^	4.6 (7.3)	2.8 (4.3)^a^
Surgery during admission, *N* (%)	64 (16.2)^a^	42 (9.4)^a^	135 (10.6)	203 (8.8)
Readmissions at seven days, *N* (%)	33 (8.3)	30 (6.7)	72 (5.9)	103 (4.5)
Mortality at seven days, *N* (%)	28 (7.1)	14 (3.1)^a^	10 (0.8)	6 (0.3)

SD: standard deviation ^a^Significantly different form the initial triage category

After reclassification, significantly more patients were admitted in the Red and Orange category (*P* < 0.001), with a significantly higher mortality in Orange (*P* = 0.028) but not in Red (*P* = 0.997). While the Yellow categories remained unchanged (mortality: *P* = 0.676, admission: *P* = 0.109), the Green category in the reclassified triage had had more patients, but the same hospital admission rates (*P* = 0.579), and mortality (*P* = 0.190). The predictive ability of the reclassified triage was significantly better than DEPT in discriminating between survivors and non-survivors at seven days; AUC (95% CI) 0.81 (0.76–0.87) vs. 0.71 (0.64–0.78), *P* < 0.001.

## Discussion

This study demonstrated that addition of suPAR to the conventional triage algorithm DEPT may improve the discriminative ability regarding in seven-day mortality in a cohort of acutely presenting medical and surgical patients. We also found a clinically transferable cut-off of 5.9 ng/ml allowing a more accurate risk stratification according to mortality risk. Also, reclassification according to the suPAR level resulted in triage categories with a better risk stratification in relation to hospital admission and mortality compared to DEPT.

The predictive abilities of suPAR were good in all triage categories apart from Red, and in accordance with previous reports of suPAR in emergency medicine, [7, 9] demonstrating a robust and high discriminative ability in relation to all-cause mortality in unselected patients arriving at the ED. The predictive ability of suPAR is equal to or better than age and other scoring systems designed to assess patients in the ED [19]. The investigated cohort consist of a large spectrum of patients arriving around the clock and suffering from both medical and surgical conditions, making this cohort suitable for testing the impact of a non-specific biomarker in relation to overall risk stratification.

However, if has not been determined whether use of a prognostic biomarker for risk stratification in the ED translates into meaningful and prognosis-changing interventions. A risk scoring system useful in emergency medicine must be simple, easily obtainable and quickly translatable to clinical decision making. One limitation of using suPAR is the need for blood sampling and analysis before the level is obtained, hence reclassification would be delayed. We used point-of-care equipment which is currently the fastest available way to analyse suPAR, however, the delay might make the suPAR level redundant in patients where treatment must be initiated immediately or within a short time frame. However, in situations with crowding, the suPAR level could be beneficial in prioritizing patients in the lower urgency classes (Yellow and Green), who might not need immediate action. This is the case of the majority of patients in the EDs and identification of those in need of a higher level of observation and faster assessment of the physician could be beneficial. This reclassification stay could potentially improve management, flow, and treatment of patients, but this must be further assessed in randomised interventional studies.

### Limitations

The study has several limitations. The analyses are done post hoc and do not include patients with gynaecological or obstetric conditions, trauma patients or patients where blood samples were not indicated. The purpose of triage algorithms is to prioritize according to urgency, however as no gold standard for urgency exists, we used surrogate endpoints. We did not have data to assess other important endpoints as time to treatment, need for antibiotic, iv fluids or imaging, which is also a limitation. Furthermore, using mortality as a measure, also includes patients with terminal illness in high urgency groups, who might not need fast assessment or continuous observation, however, patients with a very high risk of short-term mortality should ideally be assessed by a physician shortly after arrival. There was a low mortality rate at seven days, which introduce some uncertainties in the analyses. The cut-off value and subsequent reclassification was done using the same patients, and the cut-off should be validated in more cohorts of ED patients before clinical use. Another important limitation is the fact that the proportion of patients in the Red category almost doubled in the reclassified triage, which might cause flow issues and tie up personnel and resources. However, a subsequent reclassification of patients in the Red and Orange categories could be used to indicate where the most attention should be directed and ensure a high level of observation. The Danish EDs are currently using a local version of the National Early Warning Score (NEWS) for this purpose after the initial triage. It has previously be shown that addition of suPAR to NEWS improves risk prediction in both high- and low risk patients [20]. In this study, we used the Youden index for calculating the suPAR cut-off to maximize both sensitivity and specificity, as it depends on the clinical setting, whether a rule-in or a rule-out strategy is required. However, a different strategy could be use of a cut-off with the highest possible sensitivity for ruling out patients in risk of short-term mortality. Additionally, using multiple cut-offs (e.g. tertiles) might have a different impact on the reclassified triage, we chose the single cut-off for simplicity reasons making suPAR easy to implement in a future study. Patients without an available triage category were not included, which might represent a potential source of bias. Furthermore, our results might not be transferable to triage algorithms different from DEPT and the additive prognostic abilities of suPAR should be explored in addition to other algorithms as well. Finally, no randomised interventional studies to date have demonstrated that improved triage or risk scoring systems using prognostic biomarkers leads to a better prognosis. The TRIAGE III study aimed to investigate if early risk stratification using suPAR would improve patient prognosis, but found no effect on mortality, however there was no specific intervention and the suPAR level was not available at the time of triage.

## Conclusion

Addition of the prognostic biomarker suPAR to triage potentially improves prediction of seven-day mortality in the emergency department. Measurement of suPAR in relation to the triage process may allow a more accurate identification of ED patients at high and low risk of short-term mortality and enable a subsequent reclassification of patients.

## Additional file

Additional file 1: **Figure S1.** Flowchart of the TRIAGE III trial. The population included in these secondary analyses were patients arriving in the interventional periods, who had an available suPAR level and triage category. (PDF 123 kb)
