# Supplementary figures and images for: Availability of suPAR in emergency departments may improve risk stratification: a secondary analysis of the TRIAGE III trial
Source: Scand J Trauma Resusc Emerg Med. 2019 Apr 11;27:43. doi: 10.1186/s13049-019-0621-7 (PMC6458624; doi:10.1186/s13049-019-0621-7)

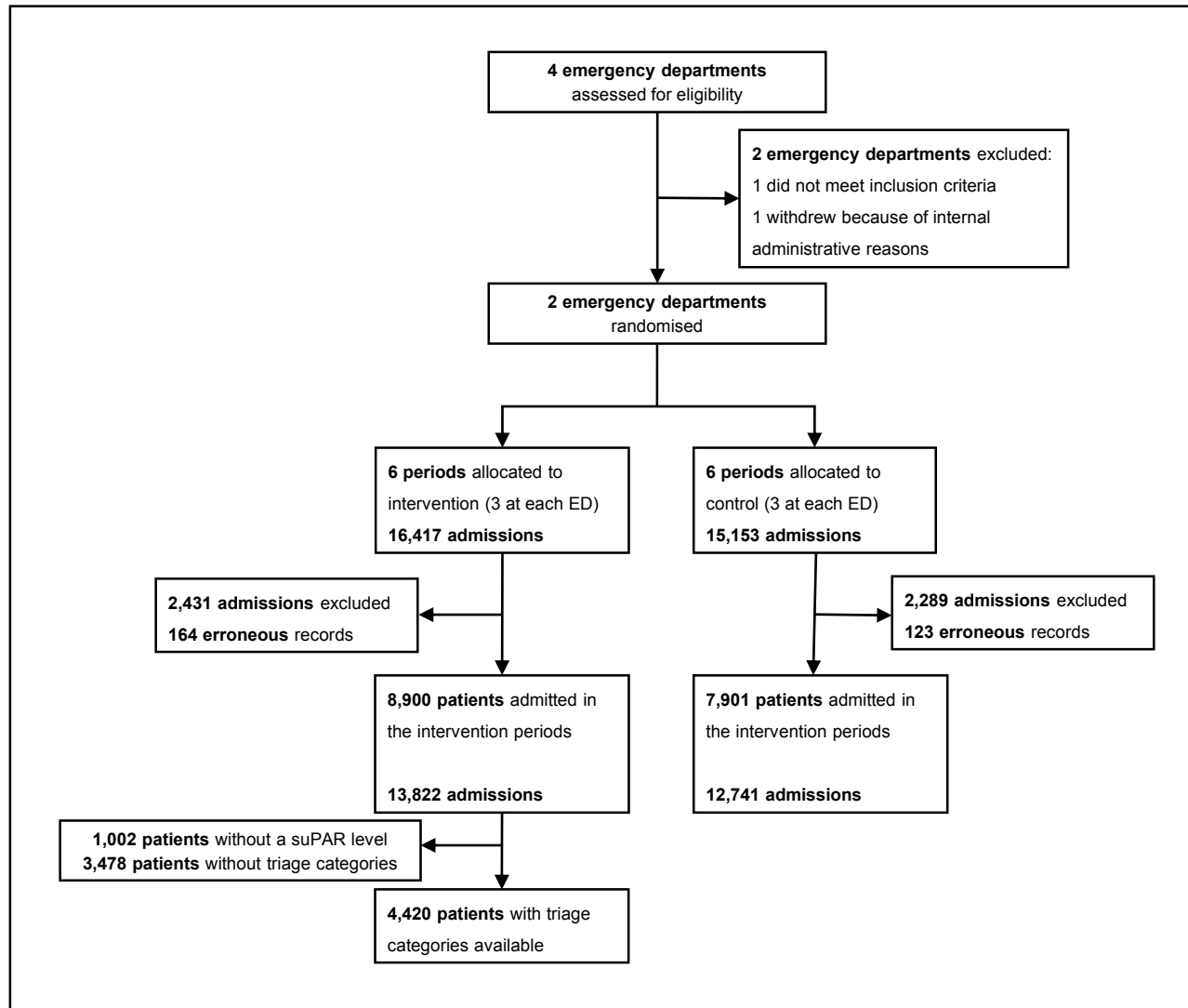

Supplement: Supplementary file 1 — Figure S1. Flowchart of the TRIAGE III trial. The population included in these secondary analyses were patients arriving in the interventional periods, who had an available suPAR level and triage category. (PDF 123 kb) [file 13049_2019_621_MOESM1_ESM.pdf]
